# Locked out of healthcare: A descriptive context of migrant health considerations in pediatrics

**DOI:** 10.1002/hpm.3490

**Published:** 2022-04-26

**Authors:** Aysha Jawed, Christine Peck

**Affiliations:** ^1^ Department of Pediatric and OB/GYN Social Work Johns Hopkins Children's Center Baltimore Maryland USA

**Keywords:** global, healthcare, immigration, migrant, paediatric

## Abstract

Over the past year, we have seen many migrant pediatric patients with significant resource limitations admitted to the Johns Hopkins Hospital. These patients are medically fragile with challenging psychosocial circumstances. They are ineligible for resources and services given their immigration status yet are in dire need of them. Our United States healthcare infrastructure is poorly designed to serve these patients. Resources are increasingly scarce, and fragmentation exists in continuity of care provided to these patients that compromises their health and safety. This global health crisis is surrounded by immense controversy especially with respect to high‐cost healthcare. Experiences from the field provide a descriptive context on the circumstances surrounding migration attributed to suboptimal access to healthcare across many developing countries. We present global health, immigration policy, and human rights implications of migration. We also propose recommendations to build a comprehensive global health network that accounts for ample disparities across healthcare systems.

## 1 DESCRIPTIVE OVERVIEW OF THE PRESENTING PROBLEM

As we actively watch the sheer magnitude of the border crisis unfold in front of our eyes whenever we tune into the news, many of us experience a range of complex emotional responses that could encompass distress, anger, sadness, disappointment, and fear among many more. As healthcare providers, we oftentimes feel more closely affected since we interface with these patients and their families on the frontline. In fact, several of these patients are very sick. During time of hospitalization, we can stabilize them acutely and their care is covered by emergency state health insurance. However for patients who will require ongoing care at home, it becomes more complicated since they are not otherwise eligible for resources and services that would be accessible to insured patients.^1^, ^2^, ^3^ The truth is that we do not have a healthcare infrastructure that can promote continuity of care for these patients.

Many of these patients have significant developmental delay and one or more medical needs that require a higher level of care at home.^1^, ^2^, ^3^ Given that they are uninsured, they are also ineligible for inpatient feeding or neurorehabilitation if warranted. Furthermore, we are oftentimes scrambling to find resources (e.g. feeding pump, cans of formula, supplemental oxygen) through donations by homecare or nonprofit organizations which are inconsistently available. Our social workers and case managers are routinely looking up price estimates for these supplies online and assessing whether families can afford these out‐of‐pocket costs. However from our frontline experiences, families cannot cover these costs. Furthermore, it can take anywhere from several months to a year and sometimes even longer for these patients to qualify for programs of the state such as Children's Medical Services (CMS). Of note, the process to apply for CMS is tedious and requires a heavy volume of paperwork with supporting documentation from a patient's family which is often challenging to obtain especially in light of resource limitations inclusive of limited support networks in the U.S. and the lingering fear of deportation.

## 2 HUMANIZING MIGRATION EXPERIENCES

Working with these patients and their families has helped us learn about their experiences and perspectives – the stressors and triggers surrounding their reasons for migration. Their narratives provide a humanistic context for the complexities involved in their decisions – the lives that they left behind, the compromises they make, the fears of the unknowns that lie ahead and much more. This firsthand information elucidates that amidst the controversies, it is crucial to take a humanistic approach with empathy and compassion when we do not know someone's story.

Of note among our migrant patients and families, none of the driving forces to migrate involved seeking economic prosperity, refuge/asylum, or educational opportunities thus far. In fact, the common denominator centred on securing needed healthcare services for their children or another family member.

### 2.1 Sequalae of trauma from crossing the border

Most migrants if not all have endured varying degrees of trauma from fleeing across the border. These travels are strenuous and can take weeks to months. Sometimes, the trauma can be marked by so much severity and depth. There can certainly be immense physical and psychological sequelae of trauma stemming from experiences during migration. In these instances, not every wound is recoverable.

In one instance, a 3‐year‐old Guatemalan toddler presented for care in our pediatric emergency department. Her parents shared that they had crossed the border to seek healthcare for her feeding and speech impairments. The child fell amidst their arduous travels across rocks, slippery surfaces, underground tunnels, uneven pathways, and deep water. She sustained injuries across several of her toes on one foot but continued bearing weight on it. Amidst persistent fears of detention and deportation, her parents decided to carry her at times when her pain was excruciating rather than seek healthcare along the way. Upon reaching their destination a week later, her parents brought her to the hospital given that three of her toes had turned black. Ultimately these toes were amputated. The long‐term medical, psychological, and developmental sequalae from this trauma early on in her life could be substantial. Of note, her clinical outcomes could have been significantly different with increased accessibility of optimal speech and feeding services in Guatemala.

### 2.2 Medication inaccessibility from corruptive practices

Corruptive practices in healthcare across many developing countries are also a trigger for migration. One infant patient's father shared that their family emigrated from Honduras given that there were no medications available for treatment in the hospitals and pharmacies in their community. Speaking with more families from Honduras and additional Central and South American countries revealed similar disclosures. Their firsthand accounts corroborated the reality that medications are routinely either confiscated by healthcare providers or pharmaceutical companies and sold for increased profits rather than administered to patients for needed treatment. The implications of this corruptive practice are colossal. In turn, more families from Central and South America are looking for medication access through seeking healthcare refuge in the U.S., knowing that its healthcare system is more equitable and accessible.

### 2.3 Medication confiscation at detention centers

Once families are detained at detention centers across border locations, they unfortunately also lose many of their belongings which can include life‐sustaining measures to maintain health. This curtailment can certainly have life‐threatening implications for migrants. In one instance, an 18‐month‐old toddler from Honduras was hospitalized with osteomyelitis. Her parents disclosed that during their travels across the border, they were detained for several days. Given an infection in her foot, she was taking antibiotics which were confiscated at the detention center and discarded. Rather than seek healthcare and risk getting detained again upon release, her parents carried her in their arms until reaching their destination a couple weeks later. Without access to antibiotics, her infection reached her bones and she developed osteomyelitis which prompted hospitalization. Her case as well as many others necessitate careful examination of inhumane treatment across detention centers especially where withheld medical treatment can result in preventable sequelae of health complications.

## 3 HEALTHCARE IMPLICATIONS

This global issue is certainly surrounded by tremendous controversy. There is significant strain on our healthcare system as financial costs incurred from increasing emergency department visits and hospitalizations among these migrant patients are on the rise. In fact, the U.S. healthcare system is not designed to serve these patients. Moreover, there is fragmentation in healthcare across local, national, and global levels which also contributes to visible health disparities across the world. Perhaps there is a divide across healthcare systems that seems impossible to bridge.

One recommendation is to potentially tap into our healthcare resources on a global level perhaps under the umbrella of the World Health Organization (WHO). It could be feasible for the WHO to create the space to promote multi‐collaborative efforts among national and international healthcare organizations, health ministries, and other public and private healthcare systems to mobilize resources and regulate provision as the basis to ensure that the supply meets the demand. Through optimizing a global healthcare network, it is certainly possible that our cross‐cultural global population may feel more well‐supported and cared for within their own national healthcare systems. It follows that developing this global alliance could help optimize equity and accessibility as well as mitigate migration attributed to suboptimal access to healthcare resources in developing countries.

## 4 HUMAN RIGHTS IMPLICATIONS

From a human rights perspective, it is imperative to ensure that safe and humane treatment of everyone is inclusive of migrants. Global support from a myriad of international human rights organizations (e.g. the United Nations) could yield more regulation and protection of all individuals at crossroads amidst countries. Existing national measures represent a limiting factor since the laws vary across countries. However, global intervention can help mitigate inhumane treatment in this context.

## 5 IMMIGRATION POLICY IMPLICATIONS

This border crisis especially in the context of healthcare warrants critical re‐examination of longstanding immigration policies as the basis to revise them to be up‐to‐date in a conscious effort to meet the surge of unmet medical needs of migrant patients in our current era. Specifically with respect to medical supplies (e.g. medications, durable medical equipment) that are part of prescribed medical treatment for these migrant patients, existing immigration policies warrant further consideration of reform to account for access to healthcare at all times and in turn ensure that these medical supplies are not confiscated at the detention centers across borders.

## 6 FINAL CONSIDERATIONS AND FUTURE DIRECTIONS

In the meantime, the question is how will we care for these patients in our healthcare systems? We recognize that undertaking a global healthcare approach will be time‐intensive, a gradual process, and necessitate obtaining support from a multitude of stakeholders.

Within our own pediatric healthcare system, our goal is to mediate this fragmentation in care through the development and implementation of an institutional approach that promotes active collaboration among several subsystems in our Hopkins healthcare system to optimize patient care outcomes and mitigate health disparities among our migrant pediatric patients. Social workers have designed a pilot integrated care model that embodies patient and family‐centered care in pediatrics and accounts for the precious value of each resource allocated in healthcare.

We know that no healthcare system is perfect and possibly is not meant to be perfect. However as voices for the voiceless, change agents, and leaders, we can each contribute towards making it accessible and equitable across humanity.

## CONFLICTS OF INTEREST

The authors have no conflicts of interest to declare that are relevant to the content of this article.

## AUTHOR CONTRIBUTIONS

Aysha Jawed conceptualized the manuscript, wrote and prepared the original draft of the manuscript, and reviewed and edited the manuscript. Christine Peck reviewed and edited the manuscript. All authors read and approved the final manuscript.

## ETHICS STATEMENT

Not applicable.

## CONSENT FOR PUBLICATION

Not applicable.

## Data Availability

Data sharing not applicable to this article as no datasets were generated or analyzed for our manuscript.
